# Metasurface-enabled on-chip multiplexed diffractive neural networks in the visible

**DOI:** 10.1038/s41377-022-00844-2

**Published:** 2022-05-27

**Authors:** Xuhao Luo, Yueqiang Hu, Xiangnian Ou, Xin Li, Jiajie Lai, Na Liu, Xinbin Cheng, Anlian Pan, Huigao Duan

**Affiliations:** 1 National Research Center for High-Efficiency Grinding, College of Mechanical and Vehicle Engineering, Hunan University, Changsha, 410082 China; 2 Institute of Precision Optical Engineering, School of Physics Science and Engineering, Tongji University, Shanghai, 200092 China; 3 Advanced Manufacturing Laboratory of Micro-Nano Optical Devices, Shenzhen Research Institute, Hunan University, Shenzhen, 518000 China; 4 2nd Physics Institute, University of Stuttgart, Pfaffenwaldring 57, 70569 Stuttgart, Germany; 5 Max Planck Institute for Solid State Research, Heisenbergstrasse 1, 70569 Stuttgart, Germany; 6 Greater Bay Area Institute for Innovation, Hunan University, Guangzhou, 511300 China

**Keywords:** Metamaterials, Imaging and sensing

## Abstract

Replacing electrons with photons is a compelling route toward high-speed, massively parallel, and low-power artificial intelligence computing. Recently, diffractive networks composed of phase surfaces were trained to perform machine learning tasks through linear optical transformations. However, the existing architectures often comprise bulky components and, most critically, they cannot mimic the human brain for multitasking. Here, we demonstrate a multi-skilled diffractive neural network based on a metasurface device, which can perform on-chip multi-channel sensing and multitasking in the visible. The polarization multiplexing scheme of the subwavelength nanostructures is applied to construct a multi-channel classifier framework for simultaneous recognition of digital and fashionable items. The areal density of the artificial neurons can reach up to 6.25 × 10^6^ mm^−2^ multiplied by the number of channels. The metasurface is integrated with the mature complementary metal-oxide semiconductor imaging sensor, providing a chip-scale architecture to process information directly at physical layers for energy-efficient and ultra-fast image processing in machine vision, autonomous driving, and precision medicine.

## Abstract (web summary)

A polarization-multiplexed metasurface-enabled diffractive neural network, which is integrated with a CMOS imaging sensor, demonstrates on-chip multi-channel sensing and multitasking in the visible.

## Graphical abstract

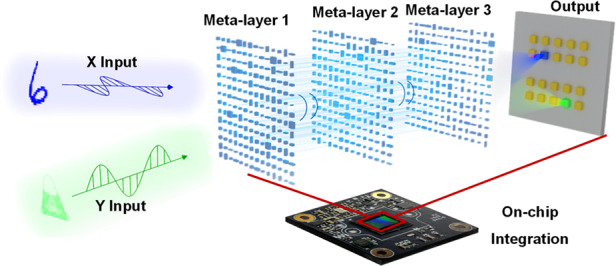

## Introduction

**Fig. 1:**
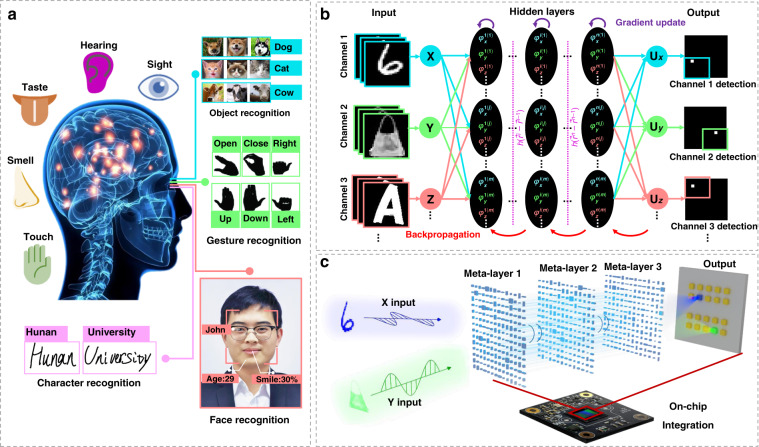
Multiplexed human brain perception system and schematic of multiplexed metasurface-based diffractive neural networks (MDNN) integrated on an imaging sensor chip. **a** Multi-channel senses of the human brain mainly comprise sight, hearing, smell, taste, and touch, among which vision can be subdivided into object recognition, gesture recognition, character recognition and face recognition, etc. **b** Architecture of the MDNN. The meta-neurons of the multiple networks are trained individually to obtain multiplexed phase distributions, which are optimized by an error backpropagation algorithm running in computer. **c** Optical layout of polarization-dependent object classification for the MDNN concept. The input is the light carrying information about the object to be recognized, e.g., a handwritten digit or a fashion product in *x*- and *y*-polarization, respectively. The hidden layers consist of polarization-multiplexed metasurfaces acting as neurons, and subsequently converge the diffraction energy to the corresponding photoelectric detection region on the CMOS chip (i.e., the output layer of network)

Artificial intelligence (AI) is a technology for simulating and extending human intelligence^1,2^, of which the artificial neural network (ANN) is one of the most widely used frameworks implemented in electronic equipment to digitally learn the representation and abstraction of data for performing advanced tasks^3,4^. ANN enables rapid performance improvement of single specific tasks, such as image recognition^5^, speech recognition^6^, and natural language processing^7^, among others^8–12^. However, the human brain works as a multi-channel system^13^ including sight, hearing, smell, taste, and touch as shown in Fig. 1a, and even each channel contains multiple sub-channels. Therefore, to achieve human-like artificial general intelligence, different capabilities should be multiplexed in a single AI system for multi-skilled AI that has wide application potential in smart homes, autonomous driving, and somatosensory interaction. Meanwhile, multiplexed AI systems can greatly increase the computing scale and degree of parallelism.

Recently, optical neural networks (ONNs)^14–20^ have attracted much attention due to their high speed, high parallelism, and low energy consumption compared with neural networks running by electrons. As a kind of ONNs, the all-optical diffractive neural networks have been proposed and experimentally demonstrated by constructing 3D printing diffractive surfaces to form a physical network^21^ at terahertz wavelengths and achieve specific functions^22–26^. Although no nonlinear activation function is applied, such multi-layer diffractive networks still exhibit a “depth” feature, i.e., the dimensionality of the transformation solution space is linearly proportional to the number of diffractive surfaces^27^. Nevertheless, the existing diffractive neural network devices, like conventional neural networks, cannot perform multiplexed information processing^28–31^. In addition, they are usually implemented in large wavelength bands with bulky sources and detectors, the advantages of all-optical computing cannot be leveraged in combination with mature image sensor chips for image processing in the optical band.

Here, we demonstrate a multiplexed metasurface-based diffractive neural network (MDNN) integrated with a complementary metal-oxide semiconductor (CMOS) imaging sensor for on-chip multi-channel sensing in the visible range. Metasurfaces are novel planar optical elements consisting of subwavelength resonators for manipulating the wavefront of light^32,33^. Optical analog computing based on ultra-thin metasurfaces attracted much attention in recent years, which enables the miniaturization of free-space and bulky systems to perform continuous mathematical operations^34^, including differentiator^35^, integrator^36^, convolutional operator^37^, and equation solver^38^, etc. Researchers also explored different degrees of freedom, such as space^39^, frequency^35,40^, and polarization^41^ to achieve parallel signal processing. However, diffractive ONNs, which are driven by matrix multiplications^19^ of discrete spatial channels, are currently not fully explored in terms of utilizing physical parametric degrees of freedom. The unprecedented ability of metasurfaces for multiparametric modulation makes them a powerful platform for multifunctional multiplexing in a single element^42–44^. We demonstrate multitasking by polarization-multiplexed metasurfaces, using a plane wave of the amplitude or phase of the object to be recognized as the input signal to achieve simultaneous recognition of digital and fashionable items. The multi-channel classifier framework is constructed by computer machine learning based on an error backpropagation approach. Due to ultra-flat and ultra-thin characteristics of metasurfaces, integration of the MDNN with CMOS chip is achieved, which provides the possibility of high-volume manufacturing in semiconductor plants with the CMOS-compatible processes. This is the first on-chip all-optical diffractive neural network realized in the visible range using metasurfaces. The areal density of neurons is greatly enhanced due to the subwavelength structure and is proportional to the number of channels.

## Results

The framework of MDNN for multiplexed classification shown in Fig. 1b comprises different types of targets to be recognized in multiple channels as inputs (e.g., handwritten digits, fashion items, letters, and so on), hidden layers with meta-neurons encoding multiplexed phases, and detectors with sub-areas for multi-channel detection. A training principle similar to that of conventional electronic neural networks is employed for each channel, which generally consists of three components: a single input layer, hidden layers with at least one layer of neurons, and a single output layer. By deep learning with error backpropagation, the multidimensional phase distributions are iteratively updated and eventually, the information from different channels converges to their specific detection regions, each corresponding to an identification class. The object can be input as an amplitude or phase component, propagated and modulated in meta-neurons. To achieve phase encoding of multiple channels for meta-neurons, we demonstrate here a kind of architecture based on polarization-multiplexed metasurfaces^45,46^ (see Fig. 1c). Note that the number of hidden layers in Fig. 1c is just for illustration, which can be any integer greater than or equal to 1. Each hidden layer consists of asymmetric meta-units, enabling the birefringence properties. By tuning the structural parameters of each meta-unit, polarization-dependent phase responses can be encoded. This allows parallel multitasking through different polarization incidence of targets. Moreover, due to the planar nature of the metasurface, it is easy to integrate it into a CMOS imaging sensor to realize an on-chip integrated AI chip.

The basic physics of the hidden layer design consisting of polarization-multiplexed meta-units is discussed. According to the Huygens–Fresnel principle^47^, each point on the wavefront can be regarded as the source of the secondary spherical wave, and the shape of the new wavefront at the next moment is determined by the envelope of the secondary spherical wave. As such, each meta-unit in a particular polarization state can be considered as a neuron (i.e., a monopole source) fully connected to the preceding and following neurons. Based on the Rayleigh–Sommerfeld diffraction integral^48^ and Jones matrix Fourier optics^49^, the optical field of (*l*+1)th layer in the all-optical meta-neurons network can be expressed as

1 $${{{\boldsymbol{U}}}}\left( {\vec r^{l + 1}} \right) = {\int} {{\int}_{ - \infty }^{ + \infty } {{{{\boldsymbol{U}}}}\left( {\vec r^l} \right) \cdot \tilde J_{{\rm{meta}}}\left( {\vec r^l} \right) \cdot h\left( {\vec r^{l + 1} - \vec r^l} \right)dxdy} }$$

where ${{{\boldsymbol{U}}}}\left( {\vec r^l} \right)$ is the optical field irradiated to the *l*th layer, and for *l* = 1, ${{{\boldsymbol{U}}}}\left( {\vec r^l} \right)$ is the projected light of the object to be identified. And $\tilde J_{{\rm{meta}}}\left( {\vec r^l} \right)$ is the Jones matrix of the birefringent metasurface of the *l*th layer, which can be expressed by $$\tilde J_{{\rm{meta}}}\left( {\vec r^l} \right) = \Gamma \left( {\theta \left( {x,y} \right)} \right)\left[ {\begin{array}{*{20}{c}} {a_x\left( {x,y} \right)e^{j\varphi _x\left( {x,y} \right)}} & 0 \\ 0 & {a_y\left( {x,y} \right)e^{j\varphi _y\left( {x,y} \right)}} \end{array}} \right]\Gamma \left( { - \theta \left( {x,y} \right)} \right)$$, which contains the complex-amplitude responses on two orthogonal axes and the orientation angle of the asymmetric structure. And $h\left( {\vec r^{l + 1} - \vec r^l} \right) = \frac{1}{{2{\uppi}}}\frac{{z^{l + 1} - z^l}}{R}\left( {\frac{1}{R} - jk} \right)\frac{{e^{jkR}}}{R}$ is the first Rayleigh–Sommerfeld impulse response function, where $R = \sqrt {\left( {x_p^{l + 1} - x_i^l} \right)^2 + \left( {y_p^{l + 1} - y_i^l} \right)^2 + \left( {z^{l + 1} - z^l} \right)^2}$ and $j = \sqrt { - 1}$. Thus, the forward propagation model of MDNN is constructed, by a cross-entropy loss function and a stochastic gradient descent approach to achieve desired output via training the network. Detailed model training and derivation are demonstrated in the “Methods” section and Supplementary Note 1.

**Fig. 2:**
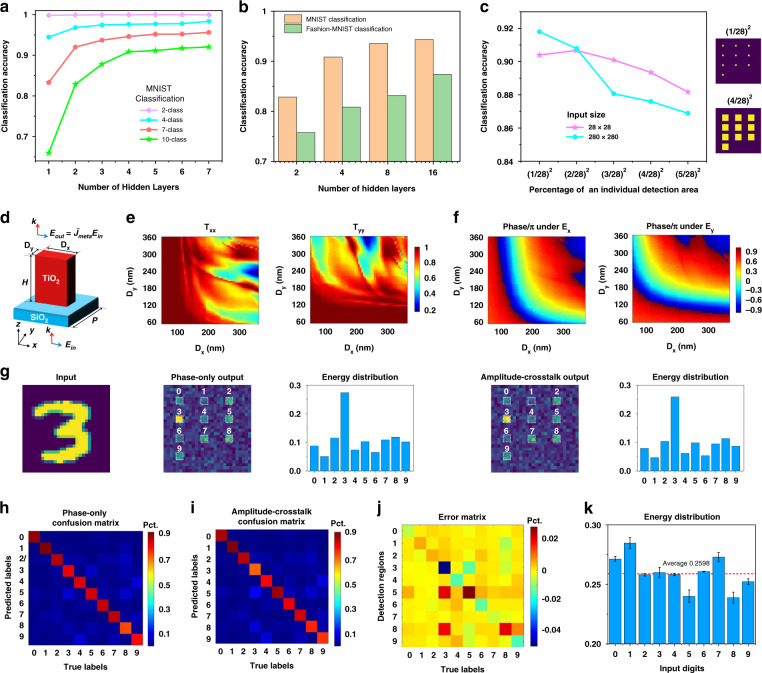
The training and design of the MDNN. **a** Variation of MNIST classification accuracy with respect to the number of hidden layers and classes. **b** Performance comparisons of MNIST and Fashion-MNIST classifiers with 10-class as an example. **c** Classification accuracy of MNIST dataset as a function of different percentage of detected regions. **d** Schematic of a single TiO_2_ meta-unit with a fixed height *H*, while tunable structure dimensions *D*_*x*_ and *D*_*y*_. Each meta-unit acts as a neuron that has multiplexed phase profiles trained by machine learning. **e**, **f** Simulated values of the transmission coefficients (*T*_*xx*_, *T*_*yy*_) and the phase shifts (*φ*_*xx*_, *φ*_*yy*_) under *x*- and *y*-polarized light, respectively. An incident wavelength of 532 nm, a nanopillar period of 400 nm, and a height of 600 nm are assumed. **g** Simulated output patterns and energy distribution percentages in the detection plane corresponding to a handwritten input of “3”, in the case of phase-only and amplitude-crosstalk networks, respectively. The confusion matrixes for **h** phase-only (amplitude is considered to be a constant value) and **i** amplitude-crosstalk MDNN are demonstrated, where the amplitude-crosstalk is based on the complex amplitude of the meta-unit in **c**. Pct. percentage. **j** Percentage error matrix between **h** and **i**. **k** Percentage of energy distribution for each digit of the network with amplitude-crosstalk. The error bars show the differences in comparison with the phase-only network

To demonstrate polarization-multiplexed MDNN for multi-channel identification, multiple sets of two-channel diffraction neural networks were trained. Two classical datasets commonly used for machine learning, Modified National Institute of Standards and Technology (MNIST)^50^ and Fashion-MNIST^51^ datasets, were exploited to demonstrate the multiplexing networks. The physical plane of the network output was divided into discrete detection regions, each representing a class of the dataset, with the region presenting the highest intensity implying the class of the object being identified. Considering the effect of the number of hidden layers as well as the number of classes classified, the networks were tested numerically using the corresponding data from 10,000 test images, which were not involved in the training, using MNIST data as an example. The variation of MNIST classification accuracy is shown in Fig. 2a, where the network has 28 × 28 neurons per layer with a period of 400 nm and a fixed layer-to-layer axial distance of 8.42 μm. It is clear that recognition accuracy generally improves as the number of hidden layers increases for larger numbers of classification inference tasks, which means the MDNN also exhibits a “depth” advantage although there is no nonlinear nature. There are also small numbers of classification cases where a single layer is competent (e.g., two-class and four-class classifications). A comparison with the 10-class example revealed that the accuracy of MNIST was slightly higher than that of Fashion-MNIST (Fig. 2b), probably because the data complexity of the former was lower than that of the latter. Figure 2c summarizes the effect of the spatial occupation of a single detection region on the classification accuracy for image inputs of 28 × 28 (for simulation) and 280 × 280 (size of meta-neurons in the experiments) pixels, taking MNIST data as an example. The neurons have a fixed period of 400 nm and the layer-to-layer axial distance of the five hidden layers with 280 × 280 × 5 neurons is 84.2 μm. It is observed that a small detection region helps to slightly improve the recognition accuracy. This will help to achieve the detection of more sub-channels within a fixed sensor area. Note that when preprocessing the input data, we scaled the input isometrically, which does not affect the amount of input information (see Supplementary Fig. S4 for more details).

As a proof-of-concept, the polarized-dependent dual-channel metasurfaces for the MDNN are designed with fixed orientations of structures as shown in Fig. 2d. The metasurface is composed of subwavelength rectangular TiO_2_ nanopillars with two independently tunable structural parameters (*D*_*x*_, *D*_*y*_) a fixed height *H*, and a period *p*. Its rectangular cross-section leads to different effective refractive indices along the two crossed axes, which is the fundamental mechanism for achieving polarization multiplexing (the experimental verification is shown in Supplementary Fig. S1). When linearly polarized light is incident along the corresponding axes, the nanopillar produces polarization-dependent phase shifts which can be expressed as a function of *D*_*x*_ and *D*_y_. The phase and amplitude under *x*- and *y*-polarization are simulated by the finite-difference time-domain (FDTD) method, where the wavelength is chosen to be 532 nm and *p* is set to 400 nm (Fig. 2e, f). The nanopillars have a height H of 600 nm without cladding to achieve a combination of multiplexed phases covering approximately two 0–2π ranges as well as a high transmittance (more details about the nanopillars with polymer cladding for multi-layer construction are in Supplementary Note 3). The detailed design methodology can be found in our previous work^46^. Since MDNN differs from conventional diffraction networks in that the meta-units introduce additional amplitude modulation, the phase-only and amplitude-crosstalk networks are compared to analyze the effect of the amplitude (Fig. 2g). Taking the handwritten digit “3” as an example (see more examples in Supplementary Fig. S5), both networks with three hidden layers can accurately redistribute the input energy to the detection region as expected. When we take the amplitude-crosstalk of the metasurface into account in the computation of the phase-only network with 10,000 handwritten digits testing dataset (the comparison of these two networks based on Fashion-MNIST is presented in Supplementary Fig. S6), the obtained recognition results have negligible error effects (Fig. 2h–j). As demonstrated in Fig. 2k, the normalized distribution of the energy in the respective target detection regions is obtained by collating all test data for handwritten digits from “0” to “9” with amplitude-crosstalk, where the error bars show the difference compared to the phase-only network. It can be seen that the average energy distribution of each target reaches more than 25%. The effect of the amplitude-crosstalk on the energy distribution is negligible, and the underlying reason is that the phase plays a major role in the modulation of light by the metasurface.

**Fig. 3:**
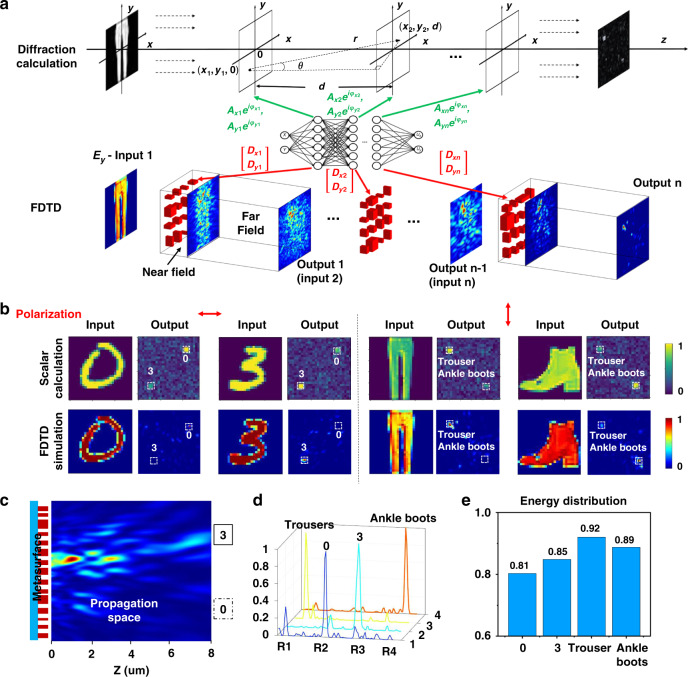
Vector simulations of multi-layer MDNN. **a** Flowcharts of scalar diffraction calculation and vector FDTD simulation. **b** Comparison of simulation results between scalar diffraction calculation and vector FDTD calculation for multi-channel classification. **c** The electric field distribution in the *z*-plane simulated by the handwritten input of “3” in **b** demonstrates that the light propagation is focused on the target region. **d** The output intensity in **b** is normalized along the *x*-direction distribution, and the maximum peaks are all confined to the detection region. R region. **e** The average energy distribution of simulated 10 groups for each of the four types of objects, all of which are randomly selected in the MNIST and Fashion-MNIST datasets, reached more than 80%

Since the metasurfaces are subwavelength arrayed devices, scalar diffraction theory is no longer applicable in principle, due to its disregard for polarization properties and inter-structural interactions. To further verify the functions of MDNN (i.e., multiple hidden layers and polarization multiplexing), we also performed a 3D full vector simulation by FDTD methods. The processes of scalar and vector simulation are compared in Fig. 3a. The scalar simulation is to calculate the light wave as a scalar quantity, which is an approximation of the actual propagation process, while the vector simulation can perfectly reproduce the interaction process between the light wave and the metasurface to obtain the information of the propagation, intensity and power. First, the input object to be detected is the amplitude or phase distribution of the polarization source; then the multi-channel diffraction phase is calculated by deep learning. For scalar simulation, the phase is directly substituted into the diffraction integral for a layer-by-layer calculation to obtain the output. For vector simulation, the phase distribution is transformed into the structural parameters of the corresponding *i*-layer metasurface array, followed by FDTD simulation to obtain the near field, and then the far field is extrapolated. If the last layer of meta-neurons calculation is completed, the output light intensity distribution is obtained. The dual-channel all-ONNs were trained based on two hidden layers, and Supplementary Fig. S7 shows the training convergence of the two-category classification from MNIST and Fashion-MNIST, indicating that both networks achieve a high accuracy rate of >99%. Note that the accuracy was obtained by blind testing the corresponding image data in the test set. Figure 3b shows the scalar diffraction calculation and vector simulation for a set of polarized-dependent dual-channel object recognition (more examples are presented in Supplementary Note 9). The light propagation in the *z*-direction from the last meta-layer to the output plane for the handwritten digit “3” is illustrated in Fig. 3c. It can be observed that the MDNN can accurately focus the input energy on the target detection region for each channel in vector simulation. Figure 3d shows the focused light field curves of the four identified objects obtained in the *x*-axis of the intercepted detection region. The peak intensities of all field intensities appear in the regions corresponding to the classified targets, in agreement with expectations. Figure 3e gives the recognition normalized energy distribution of the 10 sets of vector simulations obtained from the same simulation step, and it is obvious that the average percentage of energy for the classified targets are all higher than 80%, indicating that this FDTD vector simulation verifies the MDNN.

**Fig. 4:**
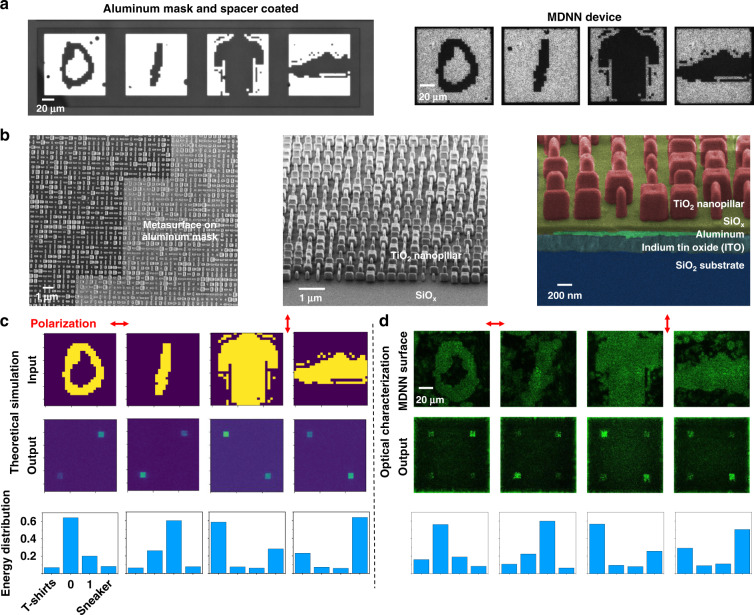
Fabrication and optical verification of the dual-channel classifier MDNN. **a** Optical micrographs of masks formed after Al deposition and coated with a spacer layer (left), and the finally fabricated MDNN devices (right). **b** Top-view (left), oblique-view (middle), and false-color cross-sectional view (right) of the scanning electron microscope (SEM) images, respectively, of the MDNN. **c** Simulation inputs and outputs of the dual-class classification MDNN under orthogonal polarization, and their energy distribution. **d** The optical demonstration of the MDNN surface (top), the output field intensity detected by a camera for these cases (middle), and energy distribution of the experiment results (bottom)

As a proof-of-concept, we first fabricated MDNN with a single hidden layer for dual-class object recognition within the double channels to verify the dual-channel neural network and study the diffraction propagation properties. The polarization-multiplexed dual-channel neural networks were trained with 280 × 280 meta-neurons (78,400 in total), and the training convergence of MNIST and Fashion-MNIST with respect to epoch number is shown in Supplementary Fig. S9a, where both networks achieved a high accuracy of greater than or equal to 99%. The accuracy is obtained from all corresponding image data in the blind test set. The phase distribution obtained after training under dual polarization is presented in Supplementary Fig. S9b. The binary Al mask was utilized as the input amplitude of the MDNN in the experiment, i.e., where the position without (with) Al structure can (cannot) transmit light with an amplitude of 1 (0). Figure 4a shows the two sets of inputs of the Al mask after adding the spacer (e.g., handwritten digits “0” and “1”, and the fashion products “t-shirts” and “sneakers”), and optical microscope images of the final fabricated MDNN device. Using SiO_*x*_ as the spacer, the surface has excellent flatness, which facilitates better subsequent exposure, deposition and etching processes to obtain high precision TiO_2_ nanopillars. The top-view, oblique-view and cross-sectional view of the scanning electron microscopy images of the MDNN device are shown in Fig. 4b, where the third one can distinguish the different layers. The Al masks, spacer, and polarization-multiplexed metasurfaces were integrated on the substrate by an electron beam lithography (EBL) overlay process (more details of the fabrication process are provided in the “Methods” section and Supplementary Note 4). To characterize the experimental performance of MDNN, we built a spatial optical path (Supplementary Fig. S10a) where different diffraction distance images can be observed to study the diffraction properties. The simulation and experimental results in Fig. 4c, d show a good agreement demonstrating the feasibility of the design and the multitasking ability of the MDNN, where the maximum energy was accurately clustered in the target detection region. The intensity of the detection region in the experiment was slightly different from that in simulation, on the one hand, because of the error of the polarizer, which cannot completely eliminate the orthogonally polarized light, and on the other hand from the fabrication error. By varying the diffraction distance (0–100 μm), the diffractive propagation properties of input light carrying different images were detected (see Supplementary Movies S1–S4). It can be found that the different input light will be gradually diffracted to a specific target region after the computing of the metasurface.

**Fig. 5:**
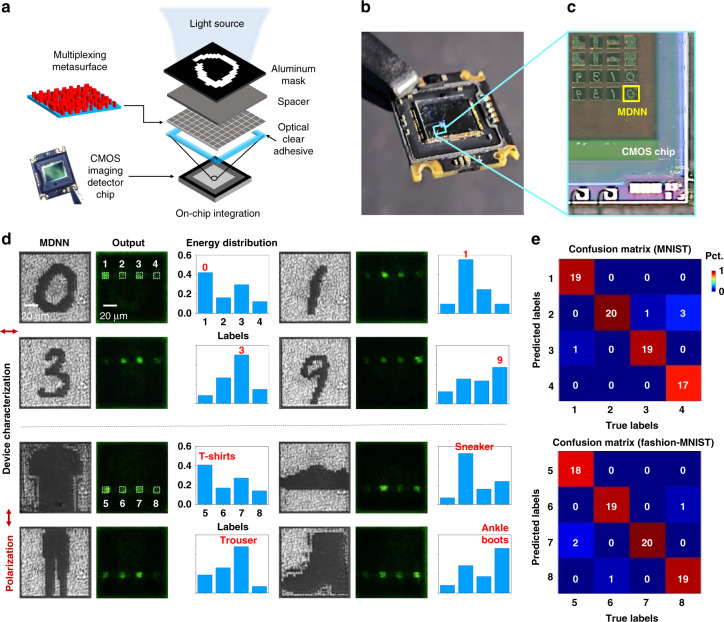
Experimental demonstration of the on-chip MDNN. **a** Exploded schematic diagram of the MDNN integrated with a CMOS chip. **b** Physical photograph of the on-chip MDNN. **c** Optical micrograph of the fabricated MDNN built on a CMOS imaging sensor. **d** The fabricated MDNN (1st and 4th columns), the output field intensity detected by the CMOS imaging sensor for these four-category classification MDNN in *x*- or *y*-polarization (2nd and 5th columns), and experimentally detected energy distribution (3rd and 6th columns). **e** Experimental confusion matrices for MNIST and Fashion-MNIST classification, respectively, with 80 images randomly selected from the correct set of simulations, for counting in the four classes (i.e., 20 per class). Pct. percentage

Next, we integrated a multiplexed MDNN capable of performing more complex recognition tasks with a commercial CMOS sensor chip to form an ultra-compact sensing and computing all-in-one chip architecture, and statistically study its recognition performance. Figure 5a–c show the schematic and physical diagrams of the on-chip MDNN. The fabricated MDNN was monolithically bonding to the CMOS imaging sensor by an optically clear adhesive (OCA) of 100-µm thickness for on-chip integration. The MDNN side of the device faces toward the CMOS chip, while the substrate faces outward, so that the distance from the MDNN to the CMOS imaging sensor, i.e., the diffraction distance, can be precisely controlled. The MDNN was designed to achieve four-category classification under each of the two-liner polarizations, containing the digits “0”, “1”, “3”, “9” and fashion products “t-shirts”, “sneaker” “trouser”, “ankle boots”, numbered from 1 to 8. The parameters of the MDNN design were the same as the previous ones except for the number of categories. In the experiments, many different sets of inputs were fabricated to systematically study the recognition performance of the device. Figure 5d reports selected examples from the experimental results of the on-chip MDNNs. We obtained the images of the CMOS output and counted the intensity distribution of each assigned detection region. It can be clearly observed that the corresponding regions get the maximum signal, proving the success of our on-chip MDNN inference capability. We selected 160 groups from the set of images that were numerically proven to be correctly classified, i.e., 20 different inputs for each category, and the statistical results of the experiments are shown in Fig. 5e. Our on-chip MDNN matches well with 93.75% and 95% between experiments and numerical simulations for digital and fashionable items, respectively. The reasons for the few target identification errors could be experimental fabrication errors, such as deviations in the overlay, and statistical errors (part of the background light affects the comparison of the intensity of the two largest detection regions).

## Discussion

We demonstrated the theoretical design and experimental implementation of a polarization-multiplexed metasurface-based all-optical linear neural network to perform various recognition tasks, such as recognizing handwritten digits and fashion items. The physical network is integrated with CMOS imaging sensors for miniaturized and portable sensing and computing all-in-one chip. Although there were also explorations of on-chip integration^26,52^, our architecture can be easily mass-produced because both CMOS chips and metasurfaces can be manufactured based on semiconductor processes. Another huge advantage of MDNN is the ability to fully exploit parallel operations of light by using the multiplexing of the metasurface. Many multiplexing schemes of the metasurface, including more polarization channel multiplexing^46^, wavelength multiplexing^53^, spatial multiplexing^54^, and vortex multiplexing^55^, can be endowed to the all-ONN to expand neural network channels. Moreover, the proposed MDNN has a subwavelength pixel size of 400 nm in the visible range, empowering the effective areal density of neurons of 6.25 × 10^6^ mm^−2^ for a single channel which will be further boosted by the combination with multiplexing. Though our fabricated on-chip MDNN has only one hidden layer, the simplest neural network, it is sufficient to demonstrate the classification of a total of eight targets within two channels (Fig. 5). To obtain higher recognition accuracy and more complex recognition characteristics, multi-layer meta-neurons can be precisely fabricated by overlay lithography^56,57^. To verify the feasibility, we have designed and simulated the multi-layer cladding metasurfaces (see Supplementary Note 3 for more details) as well as an MDNN framework with five hidden layers and 280 × 280 × 5 meta-neurons (Supplementary Fig. S4). The limitation of our linear MDNN system is that the “depth” it has is quantified by the number of linear diffraction layers, which is different from the nonlinear “depth” in the field of deep learning and signal processing. However, in the future, if the optical nonlinear activation function is incorporated into this system, it will break this limitation and bring a similar “depth” effect.

Although the current architecture has been trained to be passive and computation will be performed without additional energy input except for the power consumption of the sensor, reconfigurability is still helpful in some scenarios to achieve trainability. Mechanisms for tunable metasurfaces can be introduced into our device, such as liquid crystals^58^, phase-change^59^ materials to achieve an on-chip trainable MDNN. MDNN can currently be seen as a linear wave processor, which still has limitations for handling more complex tasks. But various optically nonlinear materials (such as nonlinear metamaterials, semiconductor materials, crystals, and doped glasses), which bring nonlinear optical effects (such as saturable absorption^60^, optical bistability^61^, and Kerr effect^62^), can be introduced into our MDNN to bring nonlinear activation functions to further enhance its convergence speed and inference ability. As an example, we employed the photorefractive crystal (SBN:60) material capable of generating nonlinear phase modulation with respect to intensity variation, to construct and simulate a nonlinear polarization-multiplexed MDNN (see Supplementary Note 12 for details). Note that the response time of nonlinear materials is much longer than the phase fluctuation time of the optical beam^63^, reducing the processing speed of nonlinear MDNNs. It is also important to note that when choosing optical nonlinear materials in MDNNs with various multiplexing mechanisms, we should be cautious to consider that the nonlinearities are decoupled from each other within individual channels. Furthermore, although isometric scaling was successful for simple datasets in our experiments, for in complex environments, we can take advantage of the structural diversity and programmability of the metasurface to construct sensing matrices that highlight task-relevant information through purposeful non-isometric scaling^64–66^. As a new class of deep learning chips for parallel processing, the pre-trained metasurface devices combined with optical imaging sensors enable to perform complex functions as simply as the human eye, and may open up a new generation of optical multi-skilled AI chips.

## Materials and methods

### Training of the MDNN

Our MDNN architectures were implemented using Python (v3.6.12) and TensorFlow (v2.1.0, Google Inc.) on a server (GeForce RTX 2080 Ti graphical processing unit (GPU, Nvidia Inc.) and Intel(R) Core (TM) i9-10980XE CPU @3.00 GHz central processing unit (CPU, Intel Inc.) with 128 GB of RAM, running the Windows 10 operating system (Microsoft)). We trained each network in the multi-channel MDNN individually, using the cross-entropy loss as a loss function, which is often used in machine learning for object classification, to maximize the signal in the target region. The neurons in each layer of the network, i.e., phases of the meta-units, are updated by a stochastic gradient descent algorithm. We used the MNIST and Fashion-MNIST datasets for training with a training batch size of 10 or 100 and a learning rate of 0.1 or 0.5. The number of neurons per layer in scalar simulations and vector simulations for training was set to be 28 × 28, while the number of neurons in a single layer in the experiments for training was set to be 280 × 280. Since we chose a relatively small learning rate and treat each training batch as an epoch, the ideal mapping function between the input and output planes was achieved after 4000, 2000, 600, and 500 epochs, respectively, and each network took about a few minutes to tens of minutes to train. Furthermore, we trained five hidden layers with 280 × 280 neurons per layer (see Supplementary Note 5). After training, the correctness of the network is verified by the Rayleigh–Sommerfeld diffraction calculation program using MATLAB.

### Sample fabrication

The MDNN sample was fabricated mainly by two processes, namely the fabrication of the metasurface and the integration with a CMOS imaging sensor, the first of which in turn consists of deposition, overlay EBL, lift-off, and atomic layer deposition (ALD), among others. First, after EBL (Raith-150^two^) patterning of a layer of polymethyl methacrylate (PMMA) resist (950 k-8%), aurum (Au) deposition and lift-off, overlay markers were defined on a quartz substrate. Subsequently, a PMMA resist layer was again coated, and after precise overlay exposure using Au markers, development, deposition, and lift-off, binary Al structure of the input signal to be identified was obtained. A 100-nm spacer protecting the Al layer was obtained by exposing hydrogen silsesquioxane. Next, the sample was coated with a 600-nm PMMA again, and the overlay marks were used to define the multiplexed meta-units pattern. After development (1 min in 1:3 MIBK:IPA solution and 1 min in IPA at −18 °C), an ALD system with TiCl_4_ precursor was used to deposit amorphous TiO_2_ onto the resist. Then, the TiO_2_ film on the top of the sample was etched by ion beam etching and the PMMA resist was stripped by reactive ion etching. Finally, we manufacture MDNNs sample on a Sony IMX686 CMOS chip with an imaging screen of 8.64 × 6.46 mm^2^ and a pixel of 0.8 μm. The most critical step in this process is to ensure that the distance between the metasurface sample and the imaging surface is sufficiently precise. Note that the diffraction distance between the hidden layers is 100 μm. Therefore, we cut an OCA with a thickness of 100 μm into the desired shape so that the metasurface is tightly bonded to the image sensor.

### Experiment setup

The experimental setup for the MDNN characterization is presented in Supplementary Fig. S10. A laser diode emitting at 532 nm (Thorlabs CPS532) was utilized as the input light. A linear polarizer was used to create the desired polarizations. The light is then directed onto the metasurface and imaged on a CMOS camera DCC3260C through a 100× objective lens. Videos of the MDNN focusing effect with diffraction distance are obtained by the movement of a stepper motor. Since the metasurface is integrated onto the CMOS imaging sensor, the output images of the experiment in Fig. 5 are collected directly by the image sensor on the CMOS chip (Sony IMX686).

## Supplementary information

Supplementary information for “Metasurface-Enabled On-Chip Multiplexed Diffractive Neural Networks in the Visible”

Movie S1

Movie S2

Movie S3

Movie S4
