# Supplementary material for: Metasurface-enabled on-chip multiplexed diffractive neural networks in the visible
Source: Light Sci Appl. 2022 May 27;11:158. doi: 10.1038/s41377-022-00844-2 (PMC9142536; doi:10.1038/s41377-022-00844-2)
Supplement: Supplementary file 1 — Supplementary information for “Metasurface-Enabled On-Chip Multiplexed Diffractive Neural Networks in the Visible” [file 41377_2022_844_MOESM1_ESM.docx]

Supplementary information for

**Metasurface-Enabled** **On-Chip Multiplexed Diffractive Neural Networks in the Visible**

Xuhao Luo^1,2,#^, Yueqiang Hu^1,3,#,*^, Xiangnian Ou^1^, Xin Li^1^, Jiajie Lai^1^, Na Liu^4,5^, Xinbin Cheng^2^, Anlian Pan^1^, and Huigao Duan^1,6,*^

1National Research Center for High-Efficiency Grinding, College of Mechanical and Vehicle Engineering, Hunan University, Changsha 410082, China

2Institute of Precision Optical Engineering, School of Physics Science and Engineering, Tongji University, Shanghai 200092, China.

3Advanced Manufacturing Laboratory of Micro-Nano Optical Devices, Shenzhen Research Institute, Hunan University, Shenzhen 518000, China

42nd Physics Institute, University of Stuttgart, Pfaffenwaldring 57, 70569 Stuttgart, Germany.

5Max Planck Institute for Solid State Research, Heisenbergstrasse 1, 70569 Stuttgart, Germany.

6Greater Bay Area Institute for Innovation, Hunan University, Guangzhou 511300, China

#These authors contributed equally.

*Corresponding authors. Email: huyq@hnu.edu.cn, [duanhg@hnu.edu.cn](mailto:duanhg@hnu.edu.cn)

**Supplementary Note 1:** **Model training and derivation of the MDNN**

We use polarization-multiplexed meta-units to construct each hidden layer. Under polarized light incidence, the meta-unit in each channel behaves like a separate neuron in a neural network, interconnected with the meta-units of the layers above and below it by diffraction of light. And under the other polarization, it behaves as another independent neuron (i.e., a unit with a different phase). Following the Rayleigh-Sommerfeld diffraction equation^1^, we can consider each neuron of a given MDNN layer as a secondary source of waves consisting of the following optical modes:

$h\left( \vec{r}^{l+1}-\vec{r}^{l} \right)=\frac{1}{2\pi}\frac{z^{l+1}-z^{l}}{R}\left( \frac{1}{R}-\mathrm{jk} \right)\frac{e^{\mathrm{jk}R}}{R}$ (S1)

where $l$represents the $l$th layer of the neural network, $k$ is the wave vector in free space, $R=\sqrt{\left( x_{p}^{l+1}-x_{i}^{l} \right)^{2}+\left( y_{p}^{l+1}-y_{i}^{l} \right)^{2}+\left( z^{l+1}-z^{l} \right)^{2}}$represents the spatial distance from the neuron located at $\left( x_{i}^{l},y_{i}^{l},z^{l} \right)$ in the $l$th layer to the neuron located at $\left( x_{p}^{l+1},y_{p}^{l+1},z^{l+1} \right)$ in the $\left( l+1 \right)$th layer, and$j =\sqrt{-1}$ .

For the $l$th layer of the network, the output function can be written as：

$u_{p}^{l+1}\text{ =}\sum_{i} h_{i,p}^{l}\text{(}x_{i}\text{,}y_{i}\text{,}z_{i}\text{)}{\cdot\tilde{J}_{meta}}_{i}^{l}\text{ (}x_{i}\text{,}y_{i}\text{,}z_{i}\text{)}{\cdot u}_{i}^{l}\text{ (}x_{i}\text{,}y_{i}\text{,}z_{i}\text{)}$ (S2)

where $u_{i}^{l}\text{ (}x_{i}\text{,}y_{i}\text{,}z_{i}\text{)}$ is the light field input to $l$th layer, $h_{i,p}^{l}\text{(}x_{i}\text{,}y_{i}\text{,}z_{i}\text{)}$ is the transfer function from $l$th layer to $\left( l+1 \right)$th layer, and $\tilde{J}_{meta}\left( \vec{r}^{l} \right)$ is the Jones matrix^2^ of the birefringent metasurface of the $l$th layer, which can be expressed by $\tilde{J}_{meta}\left( \vec{r}^{l} \right)=\Gamma\left( ϴ\left( x,y \right) \right)\left[ \begin{aligned} a_{x}{\left( x,y \right)e}^{j\varphi_{x}\left( x,y \right)}， 0 \\ 0{{， a}_{y}\left( x,y \right)e}^{j\varphi_{y}\left( x,y \right)} \end{aligned} \right]\Gamma\left( -ϴ\left( x,y \right) \right)$ , whereas $ϴ=0$.

When the input is $x$-polarized light,

${\tilde{J}_{meta}}_{X,i}^{l}\text{ }$= $\left[ \begin{aligned} {a_{X,i}^{l}\left( x,y \right)e}^{j\varphi_{X,i}^{l}\left( x,y \right)}， 0 \\ 0, {a_{Y,i}^{l}\left( x,y \right)e}^{j\varphi_{Y,i}^{l}\left( x,y \right)} \end{aligned} \right]$ $\cdot\left[ \begin{aligned} 1 \\ 0 \end{aligned} \right]$ = ${a_{X,i}^{l}\left( x,y \right)e}^{j\varphi_{X,i}^{l}\left( x,y \right)}$ (S3)

When the input is $y$-polarized light,

${\tilde{J}_{meta}}_{Y,i}^{l} = \left[ \begin{aligned} {a_{X,i}^{l}\left( x,y \right)e}^{i\varphi_{X,i}^{l}\left( x,y \right)}， 0 \\ 0, {a_{Y,i}^{l}\left( x,y \right)e}^{i\varphi_{Y,i}^{l}\left( x,y \right)} \end{aligned} \right] \cdot\left[ \begin{aligned} 0 \\ 1 \end{aligned} \right] ={a_{Y,i}^{l}\left( x,y \right)e}^{j\varphi_{Y,i}^{l}\left( x,y \right)}$ (S4)

Assuming a total of $M$-layers of metasurface, the last layer of neuron output light field is defined as:

${s_{i}^{M+1} =\left| u_{i}^{M+1} \right|}^{2}$ (S5)

The target output light field is: $g_{i}^{M+1}$.

Then, the loss function can be considered as the mean square error (MSE) between the output light field of the neurons in the last layer and the target light field, that is,

$F\left( {\tilde{J}_{meta}}_{i}^{l} \right) =\frac{1}{K}\sum_{k} \left( s_{k}^{M+1}-g_{k}^{M+1} \right)^{2}$ (S6)

Taking $x$-polarized incidence as an example (the same for the rest of the polarization states), assume that the effect of amplitude is neglected, i.e., the amplitude is agreed to be a constant, then the optimization objective is：

$min F\left( \varphi_{X,i}^{l} \right) = \frac{1}{K}\sum_{k} \left( s_{i}^{M+1}-g_{i}^{M+1} \right)^{2}, s.t. 0\leq\phi_{x,i}^{l}\leq2\pi$ (S7)

The error gradient is:

$\frac{\partial F\left( \varphi_{X,i}^{l} \right)}{\partial\varphi_{X,i}^{l}}=\frac{4}{K}\sum_{k} \left( s_{k}^{M+1}-g_{k}^{M+1} \right)\cdot real\{\left( u_{k}^{M+1} \right)^{*}\cdot\frac{\partial u_{k}^{M+1}}{\partial\varphi_{X,i}^{l}}\}$ (S8)

Where, $\frac{\partial u_{k}^{M+1}}{\partial\varphi_{X,i}^{l}}$ denotes the gradient of the output field, for the $M$th layer,

$\frac{\partial u_{k}^{M+1}\left( x_{k},y_{k},z_{k} \right)}{\partial\varphi_{X,i}^{l=M}}=\frac{\partial\sum_{k_{1}} u_{k_{1}1}^{M}\left( x_{k_{1}},y_{k_{1}},z_{k_{1}} \right)\cdot{\tilde{J}_{meta}}_{X,k1}^{M}\text{ }\left( x_{k_{1}},y_{k_{1}},z_{k_{1}} \right)\cdot h_{k_{1},k}^{M}\left( x_{k_{1}},y_{k_{1}},z_{k_{1}} \right)}{\partial\varphi_{X,i}^{l=M}}$ (S9)

Simplify to get：

$\frac{\partial u_{k}^{M+1}}{\partial\phi_{i}^{l=M}}=j{\cdot u}_{i}^{M}\cdot{\tilde{J}_{meta}}_{X,i}^{M}\cdot h_{i,k}^{M}$ (S10)

Similarly, for $\left( M-1 \right)$th layer,

$\frac{\partial u_{k}^{M+1}}{\partial\phi_{i}^{l=M-1}}=j{\cdot u}_{i}^{M-1}\cdot{\tilde{J}_{meta}}_{X,i}^{M-1}\cdot\sum_{k_{1}} h_{k_{1},k}^{M}\cdot{\tilde{J}_{meta}}_{X,k_{1}}^{M}\cdot h_{i,k_{1}}^{M-1}$ (S11)

And for $\left( M-2 \right)$th layer,

$\frac{\partial u_{k}^{M+1}}{\partial\phi_{i}^{l=M-2}}=j{\cdot u}_{i}^{M-2}\cdot{\tilde{J}_{meta}}_{X,i}^{M-2}\cdot\sum_{k_{1}} h_{k_{1},k}^{M}\cdot{\tilde{J}_{meta}}_{X,k_{1}}^{M}\cdot\sum_{k_{2}} h_{k_{2},k_{1}}^{M-1}\cdot{\tilde{J}_{meta}}_{X,k_{2}}^{M-1}\cdot h_{i,k_{2}}^{M-2}$ (S12)

And so on, the $L$th layer can be obtained,

$\frac{\partial u_{k}^{M+1}}{\partial\phi_{i}^{l=M-L}}=j{\cdot u}_{i}^{M-L}\cdot{\tilde{J}_{meta}}_{X,i}^{M-L}\cdot\sum_{k_{1}} h_{k_{1},k}^{M}\cdot{\tilde{J}_{meta}}_{X,k_{1}}^{M}\ldots\cdot\sum_{k_{2}} h_{k_{L},k_{L-1}}^{M-L+1}\cdot{\tilde{J}_{meta}}_{X,k_{L}}^{M-L+1}\cdot h_{i,k_{L}}^{M-L}$ (S13)

where $2\leq L\leq M-1$. During each iteration of error backpropagation, training data is fed into the optical neural network to generate a loss function, which is then used to update the entire neural network until the network converges. The training phase is used to obtain the network output phase distribution by summing the remainder with 2π, matching the phase of the metasurface for minimum error and considering the effect of its amplitude.

**Supplementary Note 2: Experimental verification of the polarization-multiplexed meta-neurons**

The design of the structural parameters of the polarization-multiplexed metasurface has been given in the main text, and we will verify this property experimentally in the following. Figs. S10a, b show top-view and oblique-view scanning electron microscopy (SEM) images of the fabricated TiO2 metasurface. We designed and fabricated a dual-focus metalens, which can focus the input light under x- and y-polarization onto two different focal points respectively, to verify the polarization-multiplexing, and the simulation and experimental results agree well, as shown in Figs. S1c, d.

***Fig. S1. Experimental verification of the multi-channel meta-neurons.*** *SEM images of the fabricated metasurface:* ***(a)*** *top and* ***(b)*** *side views. The scale bar is 1 μm.* ***c*** *Bifocal metasurfaces for demonstrating independent phase modulations of the designed meta-neurons under the two orthogonal polarizations.* ***d*** *Simulated and experimental results of the focal intensity field. The green arrow refers to x polarization, and the blue one refers to y polarization.*

**Supplementary Note 3: Meta-neurons with cladding**

A nanopillar with cladding is shown in Fig. S2. Unlike the structure in the main text, it has a cladding layer, which is the result of deposition without etching afterward, in order to make multilayer meta-neurons. It has a height $H$ of 1000 nm, and this relatively high thickness can be exposed by a thicker resist as well as high-pressure EBL. The rest of the parameters are the same as described in the main text. And we set the environmental refractive index to 1.4 in the simulation, which is mainly because when we want to obtain the multilayer meta-neurons by overlay EBL, we need to achieve it by multilayer adhesive, and the refractive index of adhesive is assumed to be this common parameter. It can be seen that this structure is also able to obtain a very high transmittance (close to 100% in the simulation range) and phase distribution of 0-2π. It also has significant orthogonal variability. These demonstrate that the nanopillar can be effectively applied in multilayer MDNNs.

***Fig. S2. Meta-neurons with cladding that can be used in the on-chip integration experiments with multiple hidden layers.*** *(a) Three-dimensional schematic of a single TiO_2_ meta-unit with a fixed height H, while tunable structure dimensions D_x_ and D_y_. The unit also contains a layer of PMMA cladding and a 200-nm-thickness of TiO_2_ at the top. Each meta-unit acts as a neuron that has multiplexed phase profiles trained by machine learning. (b-e) Simulated values of the transmission coefficients (*$T_{xx}$*,* $T_{yy}$*) and the phase shifts (*$\varphi_{xx}$*,*$\varphi_{yy}$*) under x- and y-polarized optical waves, respectively. An incident wavelength of 532 nm, a nanopillar period of 400nm, a height* $H$ *of 1000nm and the environmental refractive index of 1.4 are assumed.*

**Supplementary Note 4:** **Fabrication process of the on-chip MDNN**

The fabrication process is shown in Fig. S3. First, a 200-nm-thick layer of poly methyl methacrylate (PMMA) resist (950k-8%) was spin-coated onto a quartz substrate. The substrate was exposed by electron ­beam lithography (EBL), developed in -18°C environment (1 min in 1:3 MIBK:IPA solution and 1 min in IPA) after aurum (Au) deposition and lift-off process to obtain Au markers. Subsequently, a layer of PMMA with a thickness of 200 nm was spin-coated, and a layer of binary aluminum structure (i.e., input for MDNN) was obtained by accurate positioning of these Au markers through a high-precision overlay EBL exposure with the same development, thermal evaporation of metallic aluminum, and a lift-off process. Immediately, a 100 nm thickness of hydrogen silsesquioxane (HSQ) was exposed and developed (4 min in tetramethylammonium hydroxide and 1 min in deionized water) to obtain a layer of SiO_x_, in order to protect the aluminum structure. Note that HSQ is a high-resolution resist (molecular formula: HSiO_3/2_) which is converted into SiO_x_ through dehydrogenation after electron exposure and development. Then, the sample was spin-coated again with a layer of PMMA resist, with the difference that the thickness of this resist was changed to be 600 nm, in order to subsequently obtain the corresponding height of TiO_2_. After EBL exposure and the same PMMA resist development process, the cavity-shaped counter structure of the metasurface was obtained. Then, the amorphous TiO_2_ was deposited on the resist by an atomic layer deposition (ALD) system with a TiCl_4_ precursor. Then, the TiO_2_ film on the top of the sample was etched by ion beam etching (IBE) and the PMMA resist was exfoliated by reactive ion etching (RIE) to finally obtain TiO_2_ nanopillars with a high aspect ratio. Finally, the metasurface and CMOS image sensor need to be integrated, and here we use an optically clear adhesive (OCA) with a thickness of 100 μm to adhere the two together. The CMOS chip we used is Sony IMX686, and the distance between its imaging sensor and the metasurface-based MDNN is adjustable by changing the thickness of the OCA (i.e., the diffraction distance is adjustable).

***Fig. S3.*** ***Fabrication process of the on-chip MDNN.*** *(i) Exposure of the marks for* [*overlay*](javascript:;) *EBL processes. (ii) Deposition of a 30-nm-thick gold and a lift-off process to obtain gold marks. (iii) Exposure of a PMMA layer, deposition of an aluminum layer, and a lift-off process to fabricate aluminum mask. (iv) An HSQ layer was spin-coated and exposed to protect the mask. (v)* [*Overlay*](javascript:;) *EBL process on a PMMA resist layer. (vi) ALD process for depositing TiO_2_. (vi) IBE and RIE processes to remove the top layer of TiO_2_ and residual PMMA. Ultimately, the fabricated metasurface and the CMOS imaging sensor are bonded together by OCA for on-chip integration.*

**Supplementary Note 5:** **Input magnification principle and training of MDNN with multiple hidden layers**

To simplify the data preprocessing process, in all experimental parts of the main text, our network inputs are isometrically scaled by handwritten digits of 2828 pixels to obtain an input of 280280 pixels to match the number of meta-units in the experiment, as shown in Fig. S4a. It should be noted that we only change the spatial dimension of the input in this way, not the amount of information in the input, while in principle our network will increase the number and dimension of recognized information accordingly as the number of neurons increases. Fig. S4b shows the training curve of MDNN with 5 hidden layers (2802805 neurons), achieving 90.5% accuracy for the whole MNIST data (handwritten numbers from 0 to 9). Fig. S4c shows the variation of the recognition accuracy of this network as the diffraction distance changes, indicating that the diffraction distance has almost no effect on the recognition. However, it should be noted that the diffraction distance should be selected in accordance with Nyquist's sampling law to obtain a diffraction process with true and complete information. Although our main text only demonstrates a single hidden layer of MDNN for two- and four-target classification in a dual-channel, here we verify the feasibility of MDNNs for complex information recognition with multiple hidden layers. We can obtain such samples by a multilayer overlay EBL process in which the diffraction distance can be given on demand, which is a good advantage since the high accuracy distance can be guaranteed in this process. Here we have performed isometric scaling for a simple dataset like MNIST and achieved the desired measurement results. However, things in nature are complex, such as the detection of human appearance, dress, and motion status, then MDNN has to implement non-isometric scaling during information processing to distinguish task-relevant and task-irrelevant information. The metasurface can be designed into the desired structure at subwavelength scales, and in addition many modulation mechanisms can be applied to the tunability and programmability of the metasurface. Therefore, we can take advantage of the structural diversity and programmability of the metasurface by adding programmable metalenses or metasurfaces to construct the sensing matrix during detection and multiplex the scene information to the MDNN to highlight the task-relevant information.

*
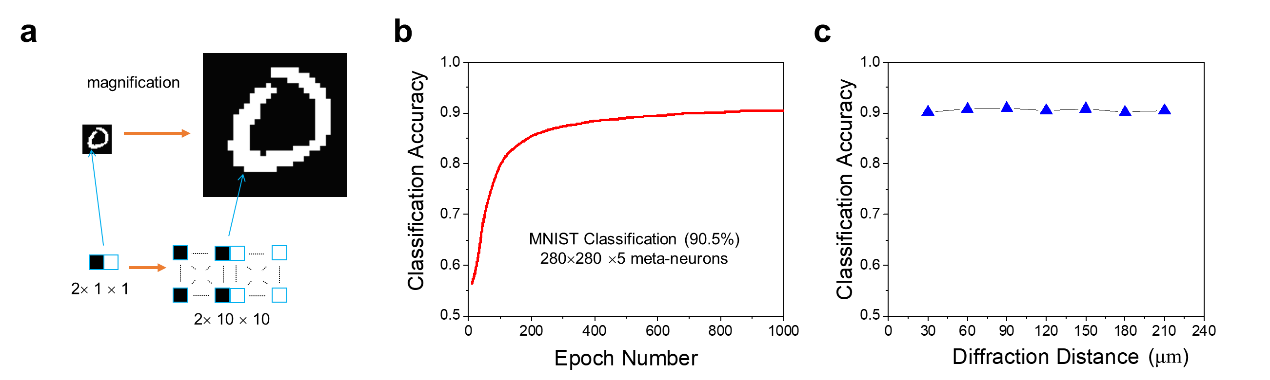
*

***Fig. S4.*** ***Input magnification principle and training of MDNN framework with 5 hidden layers.*** *(a)* *In our experiments, the input is scaled up by isometric scaling to match the number of pixels on the metasurface, but of course, it is better to use pixel-richer images. (b)* *Training convergence plot of the MDNN with five hidden layers (280×280×5 meta-neurons in total) achieves accuracy of 90.5%, taking MNIST classification as an example. (c)* *The classification accuracy of a 5-hidden-layer meta-neurons network as a function of diffraction distance.*

**Supplementary Note 6:** **Comparative examples of phase-only and amplitude-crosstalk networks**

Echoing Fig. 2g in the main text, more cases are presented in Fig. S5. Since our network is updated with phase as an adjustable parameter by error backpropagation and gradient descent algorithms, the amplitude is set as a fixed value. The metasurface, on the other hand, not only generates phase adjustment when modulating the electromagnetic wave, but also introduces the effect of amplitude crosstalk. Therefore, the crosstalk of the amplitude needs to be taken into account, as shown in Fig. 2 of the main text, and the output is obtained by substituting the obtained phase corresponding to the amplitude into the calculation. One digit each from “0” to “9” is randomly selected as input in the testing dataset. The output images and the energy distribution characteristics within the detection area are obtained for the phase-only and amplitude-crosstalk networks, respectively, and the comparison reveals that their errors are both within 4%, which are negligible. The energy is all focused within the target region, verifying that the effect of amplitude-crosstalk introduced by our MDNN is negligible, maintaining a high accuracy rate.

***Fig. S5.*** ***Comparison of output patterns and energy distribution percentages for phase-only and amplitude-crosstalk networks.*** *Some simulation comparison results are summarized with our MDNN using handwritten digits as inputs, where the inputs are randomly selected and collated from the test datasets in MNIST for the digits from “0” to “9”. For both networks, the output energy is focused in the detection area corresponding to the classification target, verifying that the amplitude of the metasurface has a weak effect on the recognition of the network (also see Fig. 2 of the main text).*

**Supplementary Note 7:** **Confusion matrix and energy distribution for MDNN based on Fashion-MNIST**

As shown in Fig. S6a, b, the confusion matrix based on the Fashion-MNIST testing dataset under phase-only and amplitude crosstalk networks, respectively, are summarized. The comparison reveals that both match 97% similarity, and only a few recognition results show deviations, among which the difference is more obvious in class 3 and class 7, but both are also below 6% error. Fig. S6c shows the percentage of the energy for the target region identified by the 10 categories based on the Fashion-MNIST testing data in the total energy of the tested regions, with an average value of 22.62%. This energy distribution result is slightly smaller than that based on the MNIST dataset in the main text, and the underlying reason is that the Fashion-MNIST has more complex features compared to the MNIST data and thus is more difficult to be identified accurately, which are acceptable within the error tolerance. Fig. S6d shows a case of fashion product recognition with sneakers as an example, where the energy under both phase-only and amplitude-crosstalk networks are focused within the target region.

***Fig. S6.*** ***Confusion matrix and energy distribution for MDNN based on Fashion-MNIST.*** *(a)* *Confusion matrix of the phase-only MDNN is shown, in which the amplitude is considered to be a constant value (e.g., 1). (b)* *The confusion matrix of the amplitude-crosstalk MDNN is shown, where the amplitudes are obtained based on the phase, to match the transmissivity of the meta-neurons. (c)* *Average energy distribution percentages for each type of fashion product for networks with amplitude-crosstalk. (d) Simulated output patterns in the detection region corresponding to a fashion product input of “sneakers”, in the case of phase-only and amplitude-crosstalk networks, respectively.*

**Supplementary Note 8:** **Training convergence plots of MDNN for vector simulations**

The classification accuracy of the MDNN gradually improves with the increase of the number of the hidden layers, as already demonstrated in Fig. 2 of the main text. An important purpose of our vector simulation is to demonstrate the possibility of implementing a polarization multiplexing diffraction neural network on the metasurface. To do a validation vector simulation while ensuring accuracy, therefore we designed a dual-target classification MDNNs under two channels. By training, as shown in Fig. S7, these networks have an accuracy of greater than 99%. Thus, we can fully substitute this trained network into FDTD for vector validation. And the obtained vector results match well with the network test results (see Fig. 3 in the main text).

***Fig. S7. MNIST and Fashion- MNIST training convergence plots of the MDNN for vector simulations.*** *The trained network has two hidden layers (28×28×2 meta-neurons in total), achieving an accuracy of 99.7% (MNIST) and 99.3% (Fashion- MNIST), respectively.*

**Supplementary Note 9:** **Comparison of results between scalar diffraction calculation and vector FDTD simulation** **with the wider detection area**

Based on the FDTD vector simulation, Fig. S8 shows the output image and energy distribution obtained for a larger detection region than that in Fig. 3b in the main text. The results show that both the large and small detection areas can accurately identify the input category. And the results of the vector simulation for the input of a handwritten digit "5" and a fashion product (i.e., trousers) in Fig. S8 are somewhat higher than the target energy distribution percentage of the network testing, which may be due to the intervention of the amplitude modulation by metasurface, but this does not affect the final recognition accuracy.

***Fig. S8.*** ***Comparison of results between scalar diffraction calculation and vector FDTD simulation for multi-channel binary classification with wider detection area.*** *The classification results show that the two simulation methods match very well, verifying the effectiveness of MDNN, and the width of the detection area has less influence on the identification.*

**Supplementary Note 10:** **Training convergence plots and phase distributions for experiments**

To experimentally validate our MDNN, an all-optical diffraction neural network with polarization-multiplexed identification was designed. The network is composed of an input layer, a single hidden layer (280280 neurons), and an output layer, which is a standard and minimalist network model. This was conceived to simplify the demonstration of our device mechanism, due to the high accuracy of this network already demonstrated in Fig. S7. The training convergence of MNIST and Fashion-MNIST as a function of the epoch number is shown in Fig. S9a, achieving the accuracy of 99% and 99.8%, respectively. This corroborates our feasibility of using this network as an experiment and that more complex object recognition can be achieved by increasing the number of hidden layers. Fig. S9b shows the phase distribution obtained after the training of this network is completed, from which it can be clearly seen that it has the phase effect of lens focusing. For x-polarized incidence, the focused phase distribution features are formed at the upper right and lower left, respectively; while for y-polarized incidence, the focused phase distribution features are formed at the upper left and lower right, respectively. This is exactly in line with our purpose of sub-regional multi-channel identification. Such a design ensures a highly accurate network while allowing multiple, non-interfering tasks to be performed simultaneously.

***Fig. S9. Network training and phase distributions for experiments.*** *(a)* *MNIST and Fashion- MNIST training convergence plots of the MDNN with one hidden layer (280×280 meta-neurons in total), achieving an accuracy of 99% and 99.8%, respectively. For each classification network, the area size of the metasurface was 112*$\times$*112 μm^2^. (b)* *The phase distributions under x- and y-polarization were obtained after training and used to produce the meta-neurons, which clearly exhibited a bifocal lens phase distribution feature.*

**Supplementary Note 11:** **Optical characterization setup for MDNN**

Fig. S10 shows the optical characteristic setup of the MDNN. In particular, Fig. S10a shows an optical test of the focusing performance of the MDNN achieved by a horizontal translation stage. A laser diode emitting at 532 nm passes through the polarizer (P) and produces x- or y- polarized light. Its irradiation on the MDNN is then passed through a 100$\times$ objective. By moving the translation stage in parallel with the lens, the light passes through a CMOS camera (DCC3260C) placed behind it to capture the videos. And the final device was made of the MDNN integrated with the CMOS image sensor, whose testing is shown in Fig. S10b. The polarized beam is focused directly on the CMOS image sensor after passing through the MDNN, and the intensity information is displayed on the computer terminal to obtain the classification results.

***Fig. S10.*** ***Optical characterization setup for MDNN.*** *(a)* *The MDNN imaging video with diffraction distance variation is obtained by moving the distance horizontally from 0 to 100 um on the translation stage. (b)* *MDNN is integrated with CMOS imaging sensors to measure imaging results for object classification.*

**Supplementary Note 12: Construction and simulation of nonlinear polarization-multiplexed MDNN**

First, we compare the framework of a diffractive deep neural network (D^2^NN) with that of an electronic neural network (ENN) in the case of a fully connected network (FCN). The standard FCN model is shown in Fig. S11a, and the relationship between the $\left( l+1 \right)$th layer and $l$th layer is $X^{l+1}=F^{l}\left( W^{l}\cdot X^{l}+B^{l} \right)$, where $W^{l}$ is the weight matrix, $B^{l}$ is the bias matrix, and $F^{l}$ is the nonlinear activation function. The weights and biases are the parameters that the network needs to learn. The common nonlinear activation functions are "sigmoid", "tanh" and "ReLU", etc. The framework of D^2^NN is shown in Fig. S11b, and its layer-to-layer relationship is $\tilde{Y^{l+1}}=\tilde{W^{l+1}}\cdot\tilde{F^{l}}\left( \tilde{Y^{l}}^{\circ}\tilde{B^{l}} \right)$, where $\tilde{W^{l+1}}$ is the weight matrix based on the free-space diffraction, $\tilde{B^{l}}= a^{l}e^{j\varphi^{l}}$ is the complex-amplitude modulated by the optical neuron, which is the parameter that the network needs to learn, $^{\circ}$ is a Hadamard product operation, and $\tilde{F^{l}}$ is the nonlinear activation function of the optics, which can be composed of materials that produce various nonlinear optical effects. In fact, this layer of nonlinear material can be arranged anywhere in the free diffraction space between the layers, and for convenience, we place it immediately behind each layer of neurons. In the FCN, the nonlinear activation function acts on the linear transformation of the input $W^{l}\cdot X^{l}+B^{l}$. While in D^2^NN, the nonlinear activation function of optics modulates the complex value $\tilde{Y^{l}}^{\circ}\tilde{B^{l}}$, i.e., a nonlinear response to amplitude or/and phase.

***Fig. S11. Comparison of the frameworks of a diffraction deep neural network (D^2^NN) and a fully connected network (FCN).*** (a) The architecture of FCN. $F^{l}$ is a nonlinear activation function such as "sigmoid" and "tanh". (b) The architecture of a nonlinear D^2^NN. $\tilde{F^{l}}$ is an optical nonlinear activation function that modulates the complex field $\tilde{Y^{l}}^{\circ}\tilde{B^{l}}$.

Fig. S12a illustrates our nonlinear MDNN, which differs most from the linear MDNN in the main text in that we add a layer of photorefractive crystals (SBN:60) after each layer of meta-neurons. The photorefractive crystal (SBN:60) is a nonlinear material whose refractive index varies with the light intensity in the medium, in other words, it generates a nonlinear phase modulation with respect to the intensity change. The source of the nonlinearity is the spatially variable light intensity, which generates free carriers through photoionization. The free carriers then change the local electric field distribution and the refractive index is eventually shifted accordingly, resulting in phase modulation. In practical applications, SBN:60 has a nonlinearity associated with the loading voltage. Therefore, considering the practical feasibility, we need to set the thickness of SBN:60 and the appropriate loading voltage to produce sufficient nonlinearity, i.e., $\Delta\varphi=2\pi\left\langle I \right\rangle/\left( 1+\left\langle I \right\rangle\right)$, where $\left\langle I \right\rangle$ is the intensity perturbation on the spatially uniform background intensity. Moreover, this nonlinear modulation is polarization insensitive. Besides, the Jones matrix of the metasurface in our MDNN is $\tilde{J}_{meta}\left( \vec{r}^{l} \right)=\Gamma\left( ϴ\left( x,y \right) \right)\left[ \begin{aligned} a_{x}{\left( x,y \right)e}^{j\varphi_{x}\left( x,y \right)}， 0 \\ 0{{， a}_{y}\left( x,y \right)e}^{j\varphi_{y}\left( x,y \right)} \end{aligned} \right]\Gamma\left( -ϴ\left( x,y \right) \right)$, whereas $ϴ=0$, and its polarization multiplexing is fully decoupled, i.e., each has a separate set of amplitudes and phases under x- or y-polarization incidence, that is, $a_{x}{\left( x,y \right)e}^{j\varphi_{x}\left( x,y \right)}$ and ${a_{y}\left( x,y \right)e}^{j\varphi_{y}\left( x,y \right)}$. In short, the photorefractive crystal (SBN:60) of $l$th layer shares the same nonlinear activation function under both polarizations of the polarization-multiplexed MDNN, which is the $\tilde{F^{l}}$ in the formula$\tilde{Y^{l+1}}=\tilde{W^{l+1}}\cdot\tilde{F^{l}}\left( \tilde{Y^{l}}^{\circ}\tilde{B^{l}} \right)$ in the D^2^NN framework. Even if we choose polarization-dependent nonlinear materials, we only need to choose $\tilde{F_{x}^{l}}$ and $\tilde{F_{y}^{l}}$ for the calculation under x- and y-polarization, respectively, when training the network, because the complex fields under different polarizations in the polarization multiplexing MDNN are independent. However, the response time of the nonlinear photorefractive crystal is much longer than the characteristic phase fluctuation time through the optical beam, which will only experience time-averaged intensity^3^. This will inevitably limit the ability to process information at high speeds in nonlinear MDNNs.

We further perform numerical simulations of the already built nonlinear polarization-multiplexed MDNN incorporating the photorefractive crystal (SBN:60), still with MINST-based classification under x-polarization and Fashion-MINST-based classification under y-polarization. The network has 5 hidden layers with a total of $28\times28\times5$ meta-neurons. Each layer of neurons is immediately followed by a layer of photorefractive crystals (SBN:60). The diffraction distance between layers is 8.42 um. As shown in Fig. S12b, after 4000 epochs, the nonlinear MDNN achieves 94.85% and 88.04% classification accuracy for MNIST and Fashion-MNIST, respectively, which is 4.17% and 5.43% improvement relative to the linear MDNN, respectively.

***Fig. S12. Numerical simulations for our nonlinear polarization-multiplexed MDNN*.** (a) Physical architecture of a nonlinear polarization-multiplexed MDNN. The nonlinear activation function is employed with a layer of photorefractive crystals (SBN:60) material. (b) The nonlinear MDNN was trained to perform the task of classifying handwritten digits and fashion products under x- and y-polarization, respectively, and accuracies were obtained as a function of epoch number, while comparing with the linear MDNN.

**References:**

1. Goodman. *Introduction to Fourier optics*. (McGraw-Hill, 1996).

2. Liu, X., Wang, B. & Guo, C. One-step Jones matrix polarization holography for extraction of spatially resolved Jones matrix of polarization-sensitive materials. *Opt. Lett.* **39**: 6170-6173 (2014).

3. Coskun, T. H., Mitchell, M., Segev, M. & Christodoulides, D. N. Theory of Incoherent Self-Focusing in Biased Photorefractive Media. *Phys. Rev. Lett.* **78**: 646-649 (1997).
